# Investigation of Meat Quality, Volatilome, and Fatty Acid Composition of Meat Parts from Liangshan Semi-Fine Wool Sheep

**DOI:** 10.3390/vetsci12060591

**Published:** 2025-06-16

**Authors:** Rui Zhang, Yongxia Xu, Hanyu Wang, Ting Bai, Xinhui Wang, Dayu Liu, Yin Zhang, Lin Zhang, Jiamin Zhang

**Affiliations:** 1 Meat Processing Key Laboratory of Sichuan Province, College of Food and Biological Engineering, Chengdu University, Chengdu 610106, China; xuyongxia999@163.com (Y.X.);; 2 Sichuan Animal Science Academy, Chengdu 610066, China

**Keywords:** Liangshan Semi-fine Wool Sheep, meat quality, fatty acid, volatilome, muscle fiber, texture

## Abstract

Liangshan Semi-fine Wool Sheep (LSWS, *Ovis aries*) are widely raised in Liangshan Yi Autonomous Prefecture, Sichuan, China. To provide a scientific basis for LSWS meat processing, our study investigated various parameters across six meat parts of LSWS including the neck, chuck roll, thin flank, outside flat, eye of round, and hind shank. Our findings revealed that thin flank displayed a higher pH_24_ compared to outside flat (*p* < 0.05), as well as greater lightness than outside flat and hind shank (*p* < 0.05), along with higher redness than eye of round (*p* < 0.05). Hardness among six meat parts ranked in descending order as chuck roll, hind shank, outside flat, eye of round, neck, and thin flank. Meanwhile, the odor activity value decreased in the order of thin flank, eye of round, hind shank, neck, chuck roll, and outside flat. In terms of the nutritional composition, hind shank exhibited the highest protein content (*p* < 0.05). Thin flank also contained elevated levels of polyunsaturated fatty acids (PUFAs, 4977 μg/g), Σn-3 (1859 μg/g) and Σn-6 (2962 μg/g) fatty acids (*p* < 0.05). Regarding human health implications, thin flank showed a lower thrombogenicity index (*p* < 0.05). This study undertook a comprehensive analysis of meat quality and nutritional attributes across six LSWS meat parts, providing a scientific foundation for LSWS meat industry development and assisting consumers in making informed purchasing decisions.

## Simple Summary

The study examined the meat quality of six meat parts of Liangshan Semi-fine Wool Sheep (LSWS) in China, analyzing parameters such as the pH, color, texture, composition, and meat odor. The findings highlighted variations in tenderness, protein and fat content, fatty acid composition, and odor attributes, with eye of round and hind shank standing out for their texture features, odor profile, and nutritional value. This research laid the groundwork for the LSWS meat industry by guiding product development based on the specific characteristics of each meat part, and it offers consumers and society valuable insights to promote healthier and more informed food consumption practices.

## 1. Introduction

**Figure 1:**
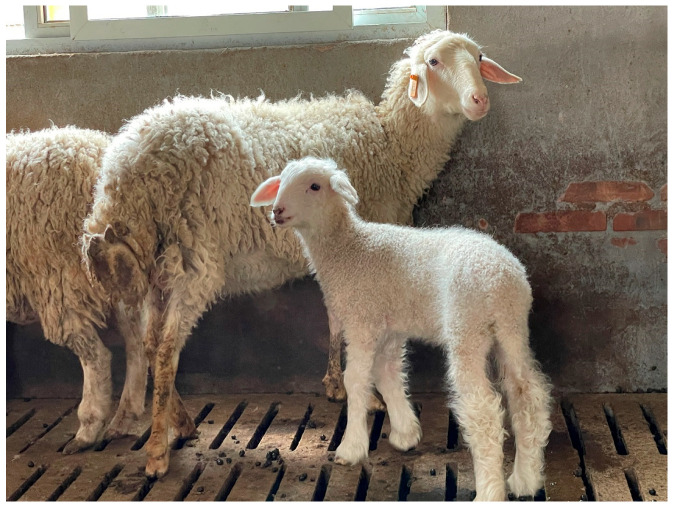
Liangshan Semi-fine Wool Sheep.

The Liangshan Semi-fine Wool Sheep (LSWS, *Ovis aries*) is a renowned dual-use breed known for its excellence in meat and wool production in Liangshan Yi Autonomous Prefecture in Sichuan Province, China. The LSWS originates as a composite breed, developed through selective breeding from a crossbreeding population that includes the local Valley-type Tibetan sheep, Xinjiang Merino sheep, Border Leicester sheep, and Romney sheep [1]. Millions of LSWS are raised, playing a crucial role in the lives of people and the local economy. Adapted to the local cold and humid climate of high mountainous regions, the LSWS exhibits a robust constitution, well-proportioned structure, and moderate size, as shown in Figure 1. Both male and female LSWS lack horns and have head hair that covers the edges of their eyes. They feature a broad chest, straight back and waist, cylindrical body shape, and an upright posture. The entire body is clothed in wool with a braided-like structure, displaying large wave-like bends, providing strong luster and uniformity. The LSWS was designated as China National Geographical Indication Agricultural Product in 2012 with the certificate number AGI00955 [2].

In China, sheep meat is typically sold as primary cuts or parts [3]. Meat quality and nutrition play a critical role in human health [4,5]. Sheep meat quality is influenced by factors such as the breed, age, feeding practice, commercial cut, fat content, and fatty acid composition [6]. Sheep breeds genetically influence attributes such as meat quality, eating quality, nutritional quality, carcass tissue distribution, and others [6]. Meats from different cuts, parts, and muscles also exhibit varied quality attributes. According to Kannan’s [7] study on Spanish does goats, the shoulder part has a much redder meat color and a higher ultimate pH compared to the loin/rib, while the loin/rib experienced less cooking loss than leg meat. Another study on Yaoshan white goats [8] indicated that the *triceps brachii* and *gluteus* meat has a lower monounsaturated fatty acid (MUFA)/polyunsaturated fatty acid (PUFA) ratio, a higher PUFA content, and a higher PUFA/saturated fatty acid (SFA) ratio than *longissimus thoracis* meat. Various studies have investigated the impact of sheep breeds and genetic backgrounds on the meat quality, flavor, and volatile compounds of different Chinese sheep breeds, including Hetian white sheep [9], small-tailed Han sheep [10], and Hu and Tan sheep [11]. Despite this, there is a notable gap in research examining the meat quality of LSWS, specifically the effects of different meat parts.

The LSWS is the first new semi-fine wool sheep breed cultivated in China. Most research efforts have focused on its wool traits [1,12]. However, in response to the declining demand in the wool market, the LSWS industry has shifted its focus from wool to meat production, and this holds new economy and research significance. To provide a scientific basis for LSWS meat processing, our study explored the meat quality, nutritional and fatty acid composition, and odor profile of six different meat parts of LSWS. This comprehensive analysis is intended to enhance meat processing practices and empower consumers to make informed purchasing decisions.

## 2. Materials and Methods

### 2.1. Animals and Sample Collection

Four 12-month-old castrated LSWS, weighing 30.40 ± 0.68 kg, were slaughtered at the LSWS Original Breed Farm in Liangshan Yi Autonomous Prefecture, Sichuan Province, China. These sheep were granted access to natural pastures and supplemented with a diet comprising corn, wheat, soybean, salt, and a mineral mix. Additionally, they were allowed access to only water 12 h before slaughter. Six meat parts, namely the neck, chuck roll, thin flank, outside flat, eye of round, and hind shank, were collected and the visible fat and connective tissues were removed. Samples for fatty acid composition analysis were promptly frozen in liquid nitrogen at the slaughterhouse and then transferred to a −80 °C freezer. Histological samples were preserved in 4% paraformaldehyde. The remaining samples, stored in ice boxes, were transported to the laboratory and kept at 4 °C for up to 24 hours (h) post-slaughter for subsequent meat quality index measurements. The slaughter process complied with the Regulation of Experimental Animals at Chengdu University (2016-4) under approval code 2020ZYD067 issued on 16 October 2020.

### 2.2. Meat Quality

#### 2.2.1. pH

The meat pH was assessed at 24 h (pH_24_) postmortem using a plug-in pH meter, Testo 205 (Testo, Lenzkirch, Germany), three times for each sample. The probe was inserted into the meat sample at a depth of 2 cm, and the average was recorded for each sample.

#### 2.2.2. Meat Color

The meat color was measured three times for each sample at 24 h postmortem using an automatic colorimeter CR-400 (Konica Minolta, Osaka, Japan) with a D65 illuminant, 8 mm aperture, and 10° observer. Prior to the measurement, calibration with a white plate was conducted. The CIE lightness (L*), redness (a*), and yellowness (b*) values of each sample were measured, based on which the average value was calculated.

#### 2.2.3. Cooking Loss

An experiment procedure described by Zhang, et al. [13] was utilized in the current work, conducted twice for each sample. Samples weighing approximately 50 g and exhibiting a similar shape were initially weighed and recorded as m1 as the weight before cooking. Subsequently, samples were placed in vacuum-sealed bags and cooked in an 80 °C water bath for 30 minutes (min). After that, samples were cooled to room temperature, and the surface moisture was dried with filter papers. The samples were reweighed and recorded as m2 as the weight after cooking. The cooking loss was calculated following the formula (m1 − m2)/m1 × 100%.

#### 2.2.4. Texture Profile Analysis (TPA)

After conducting the cooking loss procedure, a circular sampler with a diameter of 1.27 cm was used to prepare the meat sample oriented parallel to muscle fibers for TPA. The length of cylindrical sample was required to be no less than 2.5 cm. TPA was carried out once for each sample using a TA.XT plus texture analyzer (Stable Micro Systems, Surrey, UK) with a ‘V’ slot blade at a speed of 1 mm/s throughout the experiment following the China Standard [14] The results were presented as the average of four sheep samples for each meat part.

### 2.3. Nutrient Composition

The protein, fat, ash, and moisture contents of each sample were analyzed three times using China Standards [15,16,17,18]. Results were reported as percentages (%) of the meat sample.

### 2.4. Microstructure

The muscle tissue fixed in 4% paraformaldehyde underwent dehydration using an automatic dehydrator and was subsequently embedded in paraffin, which was sliced into 5 μm sections. After deparaffinization with xylene, the slices were stained with hematoxylin and eosin (H&E) solution. The slice images were captured using a BA210 digital microscope (Motic, Xiamen, China) and analyzed with ImageJ v1.53h to determine the muscle fiber density and diameter.

### 2.5. Volatile Substance Analysis

The volatile substances in raw meat samples were extracted using solid-phase microextraction and analyzed via gas chromatography-mass spectrometry (GC-MS, 7890B-5977A, Agilent Technology, Santa Clara, CA, USA). A 3 g crushed meat sample was placed in a 15 mL headspace bottle and heated at 40 °C for 20 min. GC was carried out using an HP-5MSUI column (30 m × 0.25 mm × 0.25 μm, Agilent Technology). The carrier gas helium was operated at a 1.0 mL/min flow rate and a 250 °C inlet temperature. The temperature program included an initial setting of 40 °C for 3 min; ramping up to 65 °C at 4 °C/min, holding for 4 min; a subsequent increase to 105 °C at 3 °C/min, held for 3 min; a further increase to 165 °C at 6 °C/min; and a final ramp to 230 °C at 20 °C/min. In MS, the electron ionization energy was set at 70 eV, with the temperature of the ion source and quadrupole set at 230 °C and 150 °C, respectively. The detector voltage was fixed at 350 V, and the mass scanning ranged from 40 to 550 amu (*m*/*z*).

### 2.6. Fatty Acid Analysis

#### 2.6.1. Standard Solutions

Fatty acid standard solutions involved diverse concentrations by diluting mixed standard stock solutions containing 51 fatty acids at a concentration of 4000 μg/mL into ten different levels. To construct the calibration curve, n-hexane was introduced incrementally across a spectrum ranging from 1 to 2000 μg/mL (1, 5, 10, 25, 50, 100, 250, 500, 1000 and 2000 μg/mL), encompassing a broad range of concentrations for each constituent. The linear regression equations for 51 fatty acids are shown in Table S1.

#### 2.6.2. Sample Preparation

The meat sample was extracted with the chloroform–methanol method described by Hoving, et al. [19]. Briefly, a 100 mg meat sample was mixed with 1 mL of a chloroform methanol (2:1) solution and homogenized with a tissue grinder. Then, the sample was extracted in an ultrasonic instrument for 30 min and centrifuged at 13,778× *g* for 5 min at 4 °C. The supernatant was mixed with 2 mL of a 1% sulfuric acid methanol solution for esterification in a water bath at 80 °C for 30 min and was extracted with 1 mL of n-hexane. Then, 5 mL of H_2_O (4 °C) was added and centrifuged at 13,778× *g* for 10 min at 4 °C. The supernatant was treated with 100 mg anhydrous sodium sulfate to remove excess water and was ready for GC-MS analysis.

#### 2.6.3. GC-MS

Fatty acid methyl esters (FAMEs) were analyzed on a Trace 1300 GC (Thermo Fisher Scientific, Waltham, MA, USA) using a Thermo TG-FAME column (50 m × 0.25 mm ID × 0.20 μm) with 0.63 mL/min of helium as the carrier gas. The temperatures of the injector, ion source, and interface were 250 °C, 300 °C, and 280 °C, respectively. The column temperature started with an initial 80 °C for 1 min, then increased to 160 °C at 20 °C/min and stayed for 1.5 min, then increased to 196 °C at 3 °C/min and stayed for 8.5 min, and finally increased to 250 °C at 20 °C/min and stayed for 3 min. MS was performed on an ISQ 7000 (Thermo Fisher Scientific) with the electron impact ionization mode using 70 eV of electron energy. The results were reported as the amount of fatty acids in the meat sample.

### 2.7. Nutritional Index

The nutritional index of fatty acids in different meat parts was evaluated using the formula proposed by Ulbricht and Southgate [20] as follows:

Atherogenicity index (AI) = (C12:0 + 4 × C14:0 + C16:0)/∑UFA

Thrombogenicity index (TI) = (C14:0 + C16:0 + C18:0)/[0.5 × ∑MUFA + 0.5 × ∑(n-6 PUFA) + 3 × ∑(n-3 PUFA) + ∑(n-3 PUFA)/∑(n-6 PUFA)]

### 2.8. Statistical Analysis

Data were analyzed using utilizing Prism GraphPad 9.0 software through one-way analysis of variance, followed by Tukey’s honestly significant difference test at a significance level of *p* < 0.05. The results were presented as the average and standard deviation (SD).

## 3. Results and Discussion

### 3.1. Meat pH and Color

The meat pH is linked to its palatability, tenderness, cooking loss, and meat color [21]. As depicted in Table 1, there was a significant difference in the pH value between thin flank and outside flat (*p* < 0.05). Holman, et al. [22] discovered that the difference in the pH decline between *semitendinosus* and *longissimus lumborum* muscles after slaughter could be attributable to factors such as the fiber type, glycolytic potential, and mitochondrial content. The thin flank comprised *obliquus externus abdominis* and *rectus abdominis* muscles, with around 30% type I (slow-twitch) and 70% type II (fast-twitch) muscle fibers [23]. In contrast, outside flat primarily consisted of the *biceps femoris* muscle, with approximately 17% type I and 83% type II muscle fibers [24]. Moreover, outside flat, eye of round, and hind shank muscles are crucial for sheep leg movement and presumed to exhibit high glycolytic potential and mitochondrial contents. These differences might cause the varying meat pH observed between thin flank and outside flat.

In terms of the meat color, the L* value of thin flank was significantly higher than outside flat and hind shank (*p* < 0.05). Additionally, the a* value of thin flank was significantly higher than eye of round (*p* < 0.05). No significant difference was observed in b* values among different meat parts. The meat color is one of the most intuitive factors influencing consumers purchase decisions [25]. The meat color in a well-bled carcass was primarily influenced by the myoglobin content and its various redox forms, deoxymyoglobin, oxymyoglobin, carboxymyoglobin, and metmyoglobin [26], which are closely associated with the metabolic type of muscle fibers. Research by Ithurralde, et al. [27] concerning sheep muscle fibers indicated a significant proportion (~86%) of fast type II fibers in the *semitendinosus* muscle, the main muscle in eye of round. Ibebunjo [23] reported a balanced ratio of type I, IIa, and IIb fibers (1:1:1) in goat abdominal muscles. The diverse muscle fiber composition in different meat parts might partly explain the variations in the meat color.

The hind shank exhibited significantly lower cooking loss than other LSWS meat parts (*p* < 0.05). Low cooking loss is indicative of high meat quality attributes such as tenderness, juiciness, flavor, and nutritional value. Cooking loss can vary greatly, ranging from 5.5% to 52.37%, depending on the meat type and cooking methods [28].

### 3.2. Meat Texture

The meat texture significantly influences eating behavior by affecting the breakdown of the meat structure. In this study, TPA was utilized to simulate the mastication process and assess meat palatability. Hardness, a critical factor influencing meat palatability, was notably observed, with the highest level of hardness found in chuck roll (*p* < 0.05). In contrast, neck and thin flank exhibited superior tenderness, with outside flat, eye of round, and hind shank displaying intermediate hardness levels (*p* < 0.05). A study by Starkey, et al. [29] on three primary types of sheep meat revealed that their tenderness was influenced by various factors such as soluble collagen, sarcomere length, desmin degradation, ultimate pH, intramuscular fat, and gender. Therefore, the postmortem meat aging process and macronutrient composition might lead to variations in the hardness of LSWS meat parts. Notably, the thin flank displayed the highest elasticity, whereas neck meat showed the lowest elasticity among meat parts (*p* < 0.05). Additionally, chuck roll demonstrated the greatest chewiness (*p* < 0.05). Consequently, thin flank and chuck roll, with their elevated elasticity and chewiness, resisted deformations during oral processing and necessitated more chewing before swallowing [30]. In term of resilience, hind shank and neck demonstrated the highest and lowest values, respectively (*p* < 0.05), indicating that hind shank was more resistant to chewing deformations compared to neck. Textural properties such as hardness and chewiness were found to have a negative correlation with the fat content in a cooked pork emulsion [31]. This aligned with our observation that thin flank exhibited a high fat content and the lowest levels of hardness and chewiness (*p* < 0.05). Moreover, the high fat content facilitated lubrication, resulting in a quicker eating pace [30]. While research on major lamb muscles like the *longissimus lumborum*, *biceps femoris*, *semimembranosus* and *semitendinosus* is abundant, limited data exist for meat parts like neck, chuck roll, and thin flank. Further investigation is required to explore these minor meat parts. Understanding the textural properties of LSWS meat parts could guide the development of meat processing techniques to enhance product quality and consumer satisfaction.

### 3.3. Muscle Fiber Characteristics

**Figure 2:**
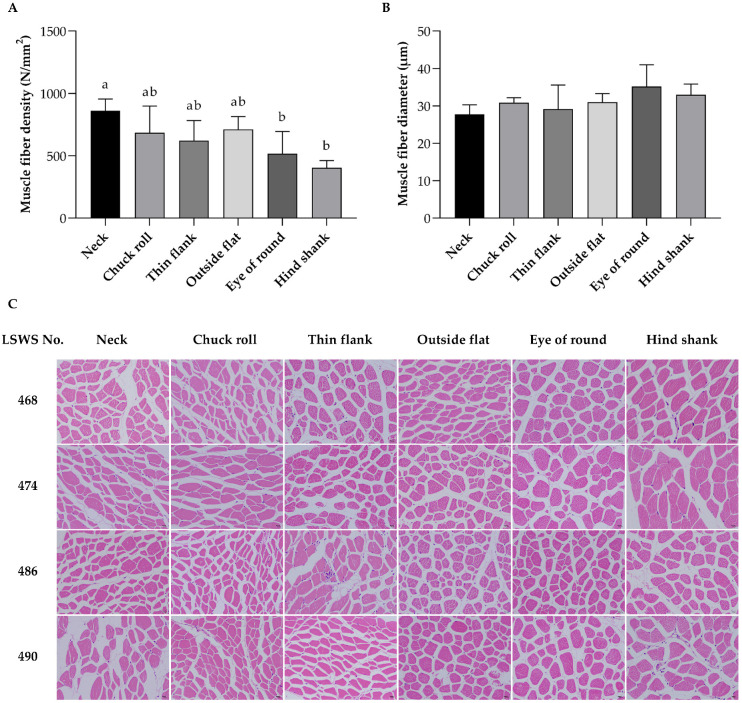
Muscle fiber characteristics of meat parts from Liangshan Semi-fine Wool Sheep. (**A**) Muscle fiber density. (**B**) Muscle fiber diameter. (**C**) H&E staining of cross-sections; bar = 10 µm. Data are shown as the average with SD, and average values sharing the same letter were not significantly different (*p* < 0.05). *n* = 4.

As depicted in Figure 2A,C, the neck meat exhibited a significantly higher muscle fiber density compared to eye of round and hind shank (*p* < 0.05). The muscle fiber diameter ranged from 28 μm in neck to 35 μm in eye of round, exhibiting no significant variation among different meat parts, shown in Figure 2B,C. The fiber density represented a crucial aspect of muscle characteristics. Previous studies have highlighted the genetic correlation between the muscle fiber density and pork quality parameters, such as the meat color and firmness [32].

### 3.4. Nutritional Composition

Table 2 illustrates that hind shank possessed the highest protein content, whereas neck exhibited the lowest protein content. The identification of hind shank as having the highest protein content suggests that it could be promoted as a preferable dietary protein source. Neck, thin flank, and eye of round exhibited a relatively high fat content among analyzed LSWS meat parts. The fat content and composition of the muscle were influenced by various factors such as the diet, adiposity, age, body weight, gender, breed, and other variables [33]. The fat content significantly influenced the meat quality and sensory attributes. A significant portion of meat flavor substances originated from the oxidation of fatty acids [34]. Drawing from life experiences, the stewed thin flank exhibited a pronounced flavor, likely stemming from its high fat content, while chuck roll and hind shank, with comparatively lower fat contents, were better suited for roasting—a traditional practice in Sichuan, China. Water constituted the largest component of fresh meat, accounting for up to 70% in LSWS meat parts. An analysis from Table 2 indicated that LSWS meat parts shared similar as and moisture contents.

### 3.5. Fatty Acid Composition

Table 3 presents a total of 51 fatty acids in six LSWS meat parts, consisting of 16 SFAs, 21 MUFAs, and 14 PUFAs. The total fatty acid content in thin flank significantly exceeded other meat parts (*p* < 0.05), while no significant difference was observed among the remaining meat parts. Eleven fatty acids in thin flank exceeded 1000 μg/g: C18:1n-7 (3399 μg/g), C18:1n-9c (2822 μg/g), C16:0 (2580 μg/g), C17:0 (2380 μg/g), C18:2n-6 (2273 μg/g), C16:1 (1964 μg/g), C14:0 (1961 μg/g), C18:0 (1899 μg/g), C18:3n-3 (1367 μg/g), C17:1 (1146 μg/g), and C18:1n-12 (1095 μg/g). The other five meat parts shared the top four most abundant fatty acids: C18:1N9C, C18:1n-7, C18:0, and C16:0, all with a concentration exceeding 1000 μg/g. These results suggested that the fatty acid composition of LSWS meat closely resembled that of pork [35] and beef [36], with C18:1, C16:0, and C18:0 emerging as the top three most abundant fatty acids. All studied LSWS meat parts displayed a similar level of C18:1n-9c. Conversely, C18:1n-7 and C18:0 exhibited the highest levels in thin flank, and their lowest levels in eye of round and hind shank, respectively (*p* < 0.05). C16:0 peaked in thin flank, while registering its lowest level in hind shank (*p* < 0.05). C18:0 and C16:0 are pivotal dietary fatty acids that significantly impact glucose and lipid metabolic disorders, as well as inflammation [37]. Therefore, the varying levels of C18:0 and C16:0 in different LSWS meat parts might contribute to their distinct health effects. For the rest of the fatty acids, thin flank had a higher level than the other five meat parts, sharing similar contents, except for C18:1n-7t. The identification of the significantly higher fatty acid content in thin flank compared to other meat parts highlighted the potential for consumers to select these meat parts with specific nutritional profiles.

Table 3 demonstrates that thin flank contained significantly higher levels of total SFAs, MUFAs, and PUFAs compared to other meat parts (*p* < 0.05). As per the guidelines jointly issued by the American Heart Association and American College of Cardiology, limiting SFA intake to 5%~6% of total daily energy intake is recommended to reduce the risk of cardiovascular disorders [38]. Therefore, the frequent consumption of thin flank might not be advisable for daily dietary practices. Epidemiological data suggested that the excessive consumption of SFAs, particularly C12:0, C14:0, and C16:0, is associated with a high risk of cardiovascular disease [39]. Thus, replacing SFAs with PUFAs has been recommend to reduce the risk of cardiovascular disease. Within LSWS meat parts, thin flank exhibited a significantly higher PUFA/SFA value than neck, chuck roll, outside flat, and hind shank (*p* < 0.05), with eye of round falling in between. Specifically, the PUFA/SFA ratios of LSWS meat parts varied from 0.26 in outside flat to 0.49 in thin flank, surpassing the PUFA/SFA ratios of 0.13 to 0.37 observed in Barbarine lamb [40], highlighting the superior nutritional value of LSWS meat. The impact of the dietary intake of n-3 and n-6 PUFAs on human metabolic disorders has been extensively reviewed [41], suggesting that a high dietary n-3 PUFA level, rather than n-6 PUFA, is linked to a lower likelihood of metabolic disorders. Furthermore, an optimal n-6/n-3 PUFA ratio, between 4:1 and 10:1, is recommended for a healthy diet [42]. In our study, thin flank exhibited a significantly higher level, up to seven times, of n-3 PUFAs and a significantly lower n-6/n-3 PUFA ratio at 2.11:1, in contrast to other meat parts with ratios ranging from 3.86 to 4.53:1 (*p* < 0.05). These findings indicated that the thin flank is a superior choice for mitigating metabolic disorders.

While the PUFA/SFA ratio serves as a general index, Ulbritcht and Southgate introduced the AI and TI to assess the atherogenic and thrombogenic potential of fatty acids for human diseases [20]. Opting for foods with low AI and TI values could help mitigate atherogenic and thrombogenic risks. The AI of sheep meat spanned from 0.49 to 1.32 in Tunisian Barbarine lamb [40], Iran Chaal lamb [43], and Italic crossbred lamb [44]. In our study, the AI across LSWS meat parts was relatively modest, ranging from 0.41 in hind shank to 0.58 in thin flank, with no significant difference observed. The TI for Tunisian Barbarine lambs varied from 1.1 to 1.15 [40], whereas the TI among LSWS meat parts was relatively low, ranging from 0.50 in thin flank to 1.10 in eye of round. In terms of TI, thin flank emerged as a preferred choice to minimize the risk of thrombogenicity. Consequently, LSWS emerged as a superior meat option due to its favorable fatty acid composition and nutritional value.

### 3.6. Volatilomes of LSWS Meat Parts

#### 3.6.1. Volatile Substances Detected in LSWS Meat Parts

A total of 38 volatile substances were identified and quantified in six LSWS meat parts and classified into six categories. Table S2 reveals that nine alcohol substances constituted the predominant portion. Within LSWS meat parts, thin flank exhibited the highest levels of four substances, namely 1-hexanol, 2-ethyl-1-hexanol, 2-propyl-1-pentanol, and (*Z*)-2-octen-1-ol (*p* < 0.05). Outside flat showcased elevated levels of 2,3-butanediol, 2-propyl-1-pentanol, and 1-octanol (*p* < 0.05). Additionally, eye of round and hind shank recorded the highest levels of (*S*)-6-methyl-1-octanol and 1-heptanol, respectively.

Among LSWS meat parts, five aldehyde substances were identified. These compounds possessed relatively low odor thresholds and interplayed with various characteristic aromatic substances, significantly contributing to meat flavor [45]. Thin flank exhibited the highest level of (*E*,*E*)-2,4-nonadienal (*p* < 0.05). The eye of round recorded elevated levels of hexanal, heptanal, and nonanal (*p* < 0.05), while hind shank showcased an elevated level of heptanal (*p* < 0.05). Hexanal, the predominant secondary product of fatty acid oxidation, contributes to the grassy and fatty flavors in sheep meat [46]. Both hexanal and nonanal were present in all studied LSWS meat parts, aligning with the findings of Zhang, et al. [47] on the characteristic odor substances of cooked mutton meatball.

Regarding ketones, esters, and ethers, hind shank exhibited the highest level of the sole detected ketone, 5-methyl-2-hexanone (*p* < 0.05). Thin flank showcased the highest levels of octyl formate and 1-(pentyloxy)-hexane (*p* < 0.05), the only detected ester and ether in the current study. Fifteen hydrocarbons were identified in LSWS meat parts, mainly generated through the homogeneous cleavage of fatty acid alkoxy radicals. Due to a high odor threshold, these hydrocarbon substances contributed minimally to the direct meat flavor but played a role in enhancing the overall meat odor profile [48].

#### 3.6.2. Odor Activity Value (OAV)

Alcohols and aldehydes stood out as the primary volatile substances responsible for the dominant odor in meat. Conversely, despite being detected at high concentrations, hydrocarbons had limited roles in shaping the meat flavor profile owing to their high odor thresholds. To evaluate the contribution of each volatile substance to the odor profile of LSWS meat, the OAV was calculated as the ratio of the concentration of volatile compounds to its threshold value. Substances with OAVs ≥ 1 significantly influenced the odor profile, while those with 1 > OAVs ≥ 0.1 made a minor contribution by coordinating the overall odor [49]. Table 4 illustrates the odor substances with OAVs ≥ 0.1 in LSWS meat parts, comprising six alcohols, five aldehydes, and one ketone.

Among LSWS meat parts, outside flat contained a higher level of 2,3-butanediol than hind shank (*p* < 0.05). Also, 2,3-butanediol has been detected in Tibetan sheep meat [50]. The thin flank displayed a higher 1-hexanol OAV compared to other LSWS meat parts (*p* < 0.05), which imparts a herbaceous, woody, and green flavor [51]. Notably, hind shank and outside flat had the highest and lowest OAV of 1-heptanol (*p* < 0.05), respectively. The presence of 1-heptanol contributes to a meat fragrance with woody and oily notes [51]. Unlike other alcohols, 1-octen-3-ol, known for its low odor threshold, was detected across all LSWS meat parts, infusing meat with mushroom, natural, and earthy odors [52]. On the contrary, (*Z*)-2-octen-1-ol was characterized by a sweet, floral, and cheese-like odor. While having a relatively low OAV, (*Z*)-2-octen-1-ol was most prominent in thin flank (*p* < 0.05). Furthermore, 1-octanol contributed intense sharp, fatty, and waxy notes to the meat [51] and displayed a low OAV with no significant differences among studied LSWS meat parts.

**Figure 3:**
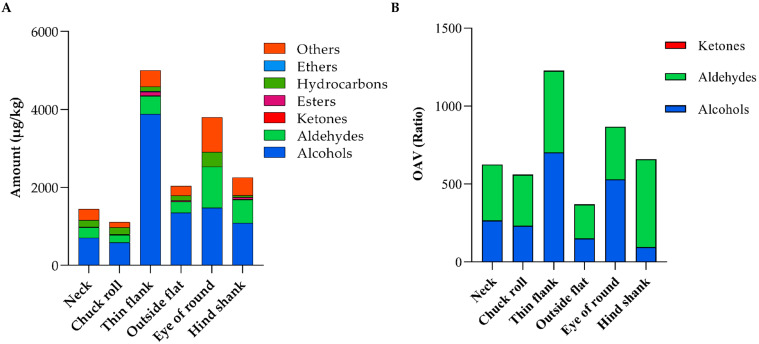
The amount (**A**) and odor activity value (**B**) of volatile substances in meat parts from Liangshan Semi-fine Wool Sheep. *n* = 4.

Among the five aldehyde odor substances, eye of round exhibited the highest OAV of hexanal (*p* < 0.05). Hexanal produces a rancid, pungent, and fresh grass flavor, contributing to a distinctive “muttony” odor [53]. Heptanal emits green, fruity, and fatty odors [54] and displayed a significantly higher OAV in eye of round and hind shank compared to other LSWS meat parts. (*E*,*E*)-2,4-Nonadienal imparts a typical meaty flavor and has been identified in Dorper lamb meat [55]. This compound had a low odor threshold and exhibited a higher OAV in neck, chuck roll, thin flank, and hind shank compared to outside flat (*p* < 0.05). Decanal yields green and fishy odors and possessed a modest OAV in thin flank and hind shank. Nonanal reveals citrus, citronella, and grass flavors [49] and serves as a crucial flavor component in mutton [56]. Nonanal was detected in all analyzed LSWS meat parts without significant variance. Consequently, the odor profile of LSWS meat was characterized by three alcohols, including 1-octen-3-ol, 1-hexanol and (*Z*)-2-octen-1-ol, alongside four aldehydes, including (*E*,*E*)-2,4-nonadienal, hexanal, nonanal, and heptanal. In summary, thin flank had the most extensive volatilomes, while chuck roll had the least, as depicted in Figure 3A. Similarly, thin flank maintained the highest OAV, shown in Figure 3B. Contrary to expectations, outside flat, not chuck roll, exhibited the lowest OAV. These olfactory attributes could guide LSWS meat product development and assist customers in making more informed purchasing decisions.

## 4. Conclusions

The LSWS is a significant contributor to sheep meat production in Liangshan Yi Autonomous Prefecture in Sichuan Province, China. LSWS meat stands out for its distinctive characteristics, as highlighted by the study conducted on the six meat parts: neck, chuck roll, thin flank, outside flat, eye of round, and hind shank. The neck and thin flank emerged as the tenderest parts, although neck had lower elastic properties than thin flank. Despite its tenderness, the neck displayed lower levels of protein and PUFA/SFA ratios. In contrast, chuck roll stood out as the toughest and chewiest meat with a low-fat content, making it less appealing to aged customers. The thin flank offered a tender yet springy and chewy texture, notable for its rich fat content, high n-3 PUFA levels, and favorable PUFA/SFA ratio. The consumption of thin flank was linked to lower thrombogenicity potential. The outside flat, eye of round, and hind shank exhibited moderate hardness levels and are commonly sliced and consumed with hot pot. Notably, outside flat and hind shank, popular sheep meat parts among Chinese customers, showed the lowest PUFA/SFA ratio. The hind shank boasted the highest protein content and lowest cooking loss. Our finding positioned eye of round and hind shank as the premium meat parts. Overall, our current work provided valuable insights that could enhance the competitiveness of the LSWS meat industry and empower consumers to make informed decisions by catering to their preferences and needs. However, further validation with a larger sample size is recommended due to the limited number of animals used in the present study.

## Figures and Tables

**Table 1:** Quality traits of meat parts from 12-month-old castrated Liangshan Semi-fine Wool Sheep.

Parameter	Neck	Chuck Roll	Thin Flank	Outside Flat	Eye of Round	Hind Shank
pH_24_	5.65 ± 0.05 ^ab^	5.67 ± 0.05 ^ab^	5.74 ± 0.01 ^a^	5.56 ± 0.03 ^b^	5.71 ± 0.15 ^ab^	5.63 ± 0.05 ^ab^
L*	43.34 ± 1.71 ^ab^	43.27 ± 1.01 ^ab^	45.75 ± 1.25 ^a^	39.97 ± 2.91 ^b^	44.04 ± 3.64 ^ab^	39.58 ± 1.17 ^b^
a*	20.89 ± 1.14 ^ab^	21.85 ± 2.04 ^ab^	22.65 ± 0.69 ^a^	19.72 ± 2.65 ^ab^	18.44 ± 1.96 ^b^	21.77 ± 1.18 ^ab^
b*	7.88 ± 1.27	8.52 ± 1.85	8.35 ± 0.89	7.53 ± 1.89	7.77 ± 0.88	7.26 ± 0.70
Cook loss (%)	31.42 ± 3.57 ^a^	32.75 ± 1.32 ^a^	31.73 ± 1.45 ^a^	32.2 ± 2.38 ^a^	33.57 ± 1.22 ^a^	26.14 ± 2.36 ^b^
Hardness (N)	7.66 ± 2.41 ^c^	26.44 ± 1.36 ^a^	6.31 ± 2.16 ^c^	15.44 ± 0.40 ^b^	14.33 ± 1.10 ^b^	15.47 ± 2.25 ^b^
Elasticity (Ratio)	0.56 ± 0.04 ^d^	0.74 ± 0.05 ^ab^	0.79 ± 0.04 ^a^	0.6 ± 0.01 ^d^	0.64 ± 0.02 ^cd^	0.69 ± 0.04 ^bc^
Chewiness (N)	3.83 ± 0.93 ^c^	11.02 ± 0.89 ^a^	3.88 ± 0.51 ^c^	5.51 ± 0.27 ^bc^	5.53 ± 0.27 ^bc^	5.76 ± 1.36 ^b^
Cohesiveness (Ratio)	0.56 ± 0.06	0.61 ± 0.01	0.57 ± 0.08	0.56 ± 0.04	0.59 ± 0.03	0.58 ± 0.07
Resilience (Ratio)	0.19 ± 0.03 ^b^	0.23 ± 0.01 ^ab^	0.21 ± 0.01 ^ab^	0.2 ± 0.02 ^ab^	0.22 ± 0.01 ^ab^	0.24 ± 0.03 ^a^

Data are shown as the average ± SD, and average values sharing the same letter were not significantly different (*p* < 0.05). *n* = 4.

**Table 2:** Nutritional composition (%) of meat parts from Liangshan Semi-fine Wool Sheep.

Nutrient	Neck	Chuck Roll	Thin Flank	Outside Flat	Eye of Round	Hind Shank
Protein	19.68 ± 1.29 ^c^	20.04 ± 0.43 ^bc^	20.03 ± 0.92 ^bc^	21.24 ± 1.16 ^abc^	22.07 ± 0.66 ^ab^	23.08 ± 1.28 ^a^
Fat	4.82 ± 1.20	2.97 ± 2.53	6.78 ± 5.45	5.10 ± 5.13	5.03 ± 2.70	0.84 ± 0.67
Ash	1.20 ± 0.12	1.25 ± 0.30	1.23 ± 0.26	1.30 ± 0.26	1.35 ± 0.25	1.01 ± 0.03
Moisture	70.62 ± 0.74	70.68 ± 0.80	71.46 ± 0.14	71.59 ± 0.77	71.3 ± 0.55	72.53 ± 1.83

Data are shown as the average ± SD, and average values sharing the same letter were not significantly different (*p* < 0.05). *n* = 4.

**Table 3:** Fatty acid composition of meat parts from Liangshan Semi-fine Wool Sheep.

Fatty Acid	Neck	Chuck Roll	Thin Flank	Outside Flat	Eye of Round	Hind Shank
C6:0	3.92 ± 1.42	3.52 ± 1.10	7.40 ± 4.20	5.31 ± 2.52	2.92 ± 0.14	2.70 ± 0.97
C8:0	3.24 ± 1.06 ^b^	3.78 ± 0.77 ^b^	27.05 ± 17.66 ^a^	2.58 ± 0.64 ^b^	2.28 ± 0.82 ^b^	1.43 ± 0.46 ^b^
C10:0	23.52 ± 10.59 ^b^	28.32 ± 2.65 ^b^	230.3 ± 169.6 ^a^	17.00 ± 8.50 ^b^	15.29 ± 5.43 ^b^	7.88 ± 2.67 ^b^
C11:0	0.47 ± 0.18 ^b^	0.62 ± 0.13 ^b^	4.51 ± 3.14 ^a^	0.31 ± 0.07 ^b^	0.28 ± 0.08 ^b^	0.22 ± 0.04 ^b^
C12:0	14.83 ± 5.69 ^b^	19.45 ± 2.79 ^b^	164.3 ± 114.8 ^a^	12.95 ± 3.01 ^b^	9.72 ± 3.96 ^b^	7.46 ± 3.82 ^b^
C13:0	1.84 ± 0.76 ^b^	2.54 ± 0.73 ^b^	34.31 ± 26.24 ^a^	1.38 ± 0.42 ^b^	0.98 ± 0.39 ^b^	0.73 ± 0.26 ^b^
C14:0	322.9 ± 178.6 ^b^	412.1 ± 52.4 ^b^	1961 ± 980 ^a^	214.8 ± 60.5 ^b^	190.3 ± 68.29 ^b^	121.1 ± 41.5 ^b^
C15:0	64.67 ± 25.61 ^b^	90.16 ± 23.44 ^b^	975.1 ± 626.0 ^a^	46.96 ± 11.38 ^b^	36.30 ± 14.53 ^b^	24.26 ± 8.60 ^b^
C16:0	2006 ± 314 ^bc^	2263 ± 191 ^ab^	2580 ± 310 ^a^	1708 ± 141 ^cd^	1636 ± 228 ^cd^	1261 ± 198 ^d^
C17:0	196.0 ± 77.4 ^b^	267.8 ± 50.8 ^b^	2380 ± 1517 ^a^	125.5 ± 21.9 ^b^	108.7 ± 31.6 ^b^	69.92 ± 24.61 ^b^
C18:0	1477 ± 305 ^ab^	1464 ± 194 ^ab^	1899 ± 653 ^a^	1402 ± 110 ^ab^	1271 ± 140 ^ab^	1046 ± 225 ^b^
C20:0	21.96 ± 5.85 ^ab^	28.21 ± 4.41 ^ab^	49.82 ± 38.10 ^a^	16.76 ± 1.34 ^b^	13.91 ± 2.81 ^b^	9.80 ± 2.17 ^b^
C21:0	1.77 ± 0.55 ^b^	2.16 ± 0.51 ^b^	25.07 ± 14.50 ^a^	1.39 ± 0.28 ^b^	1.18 ± 0.29 ^b^	0.88 ± 0.22 ^b^
C22:0	3.00 ± 1.91	3.42 ± 2.36	37.10 ± 41.26	2.62 ± 1.38	2.49 ± 1.48	1.81 ± 1.44
C23:0	1.08 ± 0.66 ^b^	1.16 ± 0.80 ^b^	8.45 ± 8.17 ^a^	0.98 ± 0.44 ^b^	0.91 ± 0.62 ^b^	0.73 ± 0.59 ^b^
C24:0	2.52 ± 0.64	3.13 ± 0.61	81.87 ± 83.21	1.97 ± 0.23	2.08 ± 0.69	2.03 ± 0.02
SFA	4145 ± 571 ^b^	4593 ± 377 ^b^	10445 ± 4125 ^a^	3556 ± 108 ^b^	3294 ± 372 ^b^	2557 ± 440 ^b^
C14:1t	23.18 ± 4.90 ^ab^	24.55 ± 2.06 ^ab^	57.20 ± 42.01 ^a^	19.21 ± 6.95 ^ab^	23.97 ± 5.86 ^ab^	17.26 ± 2.92 ^b^
C14:1	242.4 ± 30.7 ^a^	249.0 ± 13.3 ^a^	135.9 ± 62.5 ^b^	185.5 ± 69.2 ^ab^	233.0 ± 16.2 ^a^	210.0 ± 6.2 ^ab^
C15:1t	28.35 ± 14.67	22.33 ± 5.91	147.1 ± 144.7	26.09 ± 11.65	28.98 ± 11.20	14.19 ± 1.07
C15:1	11.52 ± 1.97 ^b^	13.04 ± 3.66 ^b^	36.95 ± 12.66 ^a^	9.37 ± 1.63 ^b^	10.51 ± 1.28 ^b^	7.78 ± 1.15 ^b^
C16:1t	62.35 ± 12.14 ^b^	79.07 ± 14.07 ^b^	705.7 ± 523.3 ^a^	46.75 ± 6.52 ^b^	44.63 ± 9.77 ^b^	29.02 ± 7.97 ^b^
C16:1	242.9 ± 70.5 ^b^	306.5 ± 40.0 ^b^	1964 ± 1197 ^a^	189.4 ± 33.7 ^b^	174.5 ± 30.4 ^b^	143.0 ± 38.4 ^b^
C17:1t	30.39 ± 7.00 ^b^	37.59 ± 5.66 ^b^	185.5 ± 116.0 ^a^	22.79 ± 3.54 ^b^	22.25 ± 5.45 ^b^	16.26 ± 2.36 ^b^
C17:1	95.18 ± 24.96 ^b^	122.2 ± 16.1 ^b^	1146 ± 959 ^a^	69.38 ± 11.86 ^b^	67.06 ± 15.32 ^b^	46.51 ± 1.34 ^b^
C18:1n-12 t	25.41 ± 11.15 ^b^	23.01 ± 5.84 ^b^	84.39 ± 58.88 ^a^	21.96 ± 9.32 ^b^	21.44 ± 9.81 ^b^	11.70 ± 2.32 ^b^
C18:1n-9 t	42.07 ± 4.68 ^b^	51.1 ± 11.77 ^b^	102.0 ± 27.5 ^a^	36.85 ± 11.70 ^b^	29.88 ± 3.75 ^b^	25.98 ± 1.08 ^b^
C18:1n-7 t	141.7 ± 37.4 ^bc^	176.8 ± 43.7 ^ab^	230.8 ± 9.9 ^a^	126.7 ± 19.6 ^bcd^	89.11 ± 31.66 ^cd^	66.73 ± 30.71 ^d^
C18:1n-12	81.84 ± 50.28 ^b^	75.31 ± 14.16 ^b^	1095 ± 262 ^a^	42.32 ± 9.31 ^b^	39.97 ± 15.89 ^b^	26.66 ± 9.37 ^b^
C18:1n-9 c	2586 ± 467	2573 ± 352	2822 ± 882	2389 ± 260	2219 ± 46	1910 ± 138
C18:1n-7	1740 ± 583 ^bc^	2259 ± 137 ^b^	3399 ± 324 ^a^	1363 ± 590 ^bc^	1189 ± 631 ^c^	1320 ± 76 ^bc^
C19:1n-12 t	14.90 ± 3.91 ^b^	18.13 ± 4.12 ^b^	304.9 ± 284.1 ^a^	11.71 ± 2.79 ^b^	11.87 ± 3.84 ^b^	8.90 ± 2.02 ^b^
C19:1n-9 t	28.17 ± 5.86 ^b^	34.52 ± 7.45 ^b^	274.6 ± 127.1 ^a^	22.11 ± 0.50 ^b^	21.49 ± 5.55 ^b^	16.29 ± 4.63 ^b^
C20:1t	20.36 ± 5.38 ^b^	20.18 ± 4.10 ^b^	64.85 ± 34.47 ^a^	16.27 ± 3.56 ^b^	18.14 ± 3.07 ^b^	15.12 ± 0.14 ^b^
C20:1	61.46 ± 8.96 ^b^	77.94 ± 25.41 ^b^	270.0 ± 145.9 ^a^	44.51 ± 8.12 ^b^	45.46 ± 8.73 ^b^	31.69 ± 6.93 ^b^
C22:1n-9 t	11.11 ± 2.30 ^b^	11.68 ± 1.31 ^b^	31.78 ± 20.30 ^a^	9.79 ± 1.17 ^b^	10.72 ± 1.68 ^b^	8.10 ± 1.64 ^b^
C22:1n-9	14.74 ± 1.94 ^ab^	15.31 ± 1.63 ^ab^	113.5 ± 108.3 ^a^	13.11 ± 2.00 ^b^	11.55 ± 1.60 ^b^	9.01 ± 0.97 ^b^
C24:1	18.02 ± 6.38 ^ab^	17.94 ± 5.36 ^ab^	46.45 ± 31.8 ^a^	15.97 ± 3.62 ^ab^	16.44 ± 5.33 ^ab^	12.66 ± 2.21 ^b^
MUFA	5522 ± 414 ^b^	61899 ± 619 ^b^	13139 ± 4077 ^a^	4676 ± 536 ^b^	3774 ± 1668 ^b^	3591 ± 673 ^b^
C18:2n-6 t	22.31 ± 3.56 ^b^	31.06 ± 7.07 ^b^	125.9 ± 81.4 ^a^	17.15 ± 3.20 ^b^	15.52 ± 4.26 ^b^	11.53 ± 2.37 ^b^
C18:2n-6	684.5 ± 172.2 ^b^	801.7 ± 299.2 ^b^	2273 ± 652 ^a^	522.3 ± 159.1 ^b^	563.4 ± 121.4 ^b^	389.9 ± 242.4 ^b^
C18:3n-6	8.36 ± 1.52 ^b^	10.02 ± 2.73 ^b^	79.14 ± 51.94 ^a^	6.75 ± 1.55 ^b^	7.62 ± 0.93 ^b^	5.06 ± 2.19 ^b^
C18:3n-3	99.44 ± 34.84 ^b^	132.6 ± 55.7 ^b^	1367 ± 771 ^a^	73.31 ± 18.56 ^b^	71.61 ± 19.19 ^b^	47.41 ± 22.55 ^b^
C20:2	8.23 ± 1.95 ^b^	10.38 ± 3.82 ^b^	65.73 ± 51.51 ^a^	6.94 ± 1.96 ^b^	6.94 ± 1.35 ^b^	5.69 ± 1.68 ^b^
C20:3n-6	18.80 ± 6.22 ^b^	15.44 ± 1.19 ^b^	62.58 ± 30.10 ^a^	17.59 ± 6.39 ^b^	22.04 ± 0.45 ^b^	14.63 ± 8.93 ^b^
C20:3n-3	3.65 ± 0.65 ^b^	4.18 ± 1.08 ^b^	48.24 ± 41 ^a^	3.25 ± 0.41 ^b^	2.72 ± 0.45 ^b^	2.30 ± 0.39 ^b^
C20:4n-6	226.8 ± 46.9 ^b^	223.1 ± 70.7 ^b^	399.4 ± 81.1 ^a^	175.2 ± 55.1 ^b^	274.6 ± 67.7 ^ab^	180.4 ± 82.5 ^b^
C22:2	3.32 ± 0.86	3.20 ± 0.60	20.23 ± 22.07	3.33 ± 0.67	3.01 ± 0.52	2.43 ± 0.79
C20:5n-3	22.70 ± 6.14 ^b^	19.48 ± 2.39 ^b^	60.55 ± 26.53 ^a^	27.89 ± 16.35 ^ab^	44.52 ± 12.78 ^ab^	18.33 ± 13.97 ^b^
C22:4	19.16 ± 1.22 ^b^	24.10 ± 10.27 ^b^	69.62 ± 46.54 ^a^	15.23 ± 2.66 ^b^	20.37 ± 5.54 ^b^	16.23 ± 3.38 ^b^
C22:5n-6	7.84 ± 1.67 ^b^	8.84 ± 2.63 ^ab^	23.14 ± 15.69 ^a^	6.37 ± 2.33 ^b^	8.84 ± 3.13 ^ab^	5.56 ± 1.83 ^b^
C22:5n-3	82.81 ± 18.67 ^b^	77.26 ± 3.85 ^b^	344.6 ± 229.3 ^a^	70.10 ± 27.66 ^b^	102.5 ± 28.4 ^b^	59.48 ± 27.94 ^b^
C22:6n-3	9.46 ± 4.31 ^b^	11.14 ± 5.62 ^ab^	39.10 ± 28.20 ^a^	8.02 ± 6.68 ^b^	14.15 ± 6.48 ^ab^	5.42 ± 0.46 ^b^
PUFA	1217 ± 290 ^b^	1349 ± 412 ^b^	4977 ± 1676 ^a^	909.6 ± 249.7 ^b^	1152 ± 255 ^b^	763.0 ± 398.8 ^b^
PUFA/SFA	0.30 ± 0.07 ^b^	0.30 ± 0.1 ^b^	0.49 ± 0.08 ^a^	0.26 ± 0.07 ^b^	0.35 ± 0.05 ^ab^	0.29 ± 0.12 ^b^
C18:3n-3	99.44 ± 34.84 ^b^	132.6 ± 55.71 ^b^	1367 ± 771 ^a^	73.31 ± 18.56 ^b^	71.61 ± 19.19 ^b^	47.41 ± 22.55 ^b^
C20:3n-3	3.65 ± 0.65 ^b^	4.18 ± 1.08 ^b^	48.24 ± 41.00 ^a^	3.25 ± 0.41 ^b^	2.72 ± 0.45 ^b^	2.30 ± 0.39 ^b^
C20:5n-3	22.70 ± 6.14 ^b^	19.48 ± 2.39 ^b^	60.55 ± 26.53 ^a^	27.89 ± 16.35 ^ab^	44.52 ± 12.78 ^ab^	18.33 ± 13.97 ^b^
C22:5n-3	82.81 ± 18.67 ^b^	77.26 ± 3.85 ^b^	344.6 ± 229.3 ^a^	70.10 ± 27.66 ^b^	102.5 ± 28.4 ^b^	59.48 ± 27.94 ^b^
C22:6n-3	9.46 ± 4.31 ^b^	11.14 ± 5.62 ^ab^	39.10 ± 28.20 ^a^	8.02 ± 6.68 ^b^	14.15 ± 6.48 ^ab^	5.42 ± 0.46 ^b^
∑n-3	218.1 ± 63.0 ^b^	225.3 ± 30.3 ^b^	1859 ± 1054 ^a^	182.6 ± 66.8 ^b^	235.5 ± 61.2 ^b^	131.6 ± 58.8 ^b^
C18:2n-6t	22.31 ± 3.56 ^b^	31.06 ± 7.07 ^b^	125.9 ± 81.4 ^a^	17.15 ± 3.20 ^b^	15.52 ± 4.26 ^b^	11.53 ± 2.37 ^b^
C18:2n-6	684.5 ± 172.2 ^b^	801.7 ± 299.2 ^b^	2272 ± 652 ^a^	522.3 ± 159.1 ^b^	563.4 ± 121.4 ^b^	389.9 ± 242.4 ^b^
C18:3n-6	8.36 ± 1.52 ^b^	10.02 ± 2.73 ^b^	79.14 ± 51.94 ^a^	6.75 ± 1.55 ^b^	7.62 ± 0.93 ^b^	5.06 ± 2.19 ^b^
C20:3n-6	18.80 ± 6.22 ^b^	15.44 ± 1.19 ^b^	62.58 ± 30.10 ^a^	17.59 ± 6.39 ^b^	22.04 ± 0.45 ^b^	14.63 ± 8.93 ^b^
C20:4n-6	226.8 ± 46.9 ^b^	223.1 ± 70.7 ^b^	399.4 ± 81.1 ^a^	175.2 ± 55.1 ^b^	274.6 ± 67.7 ^ab^	180.4 ± 82.5 ^b^
C22:5n-6	7.84 ± 1.67 ^b^	8.84 ± 2.63 ^ab^	23.14 ± 15.69 ^a^	6.37 ± 2.33 ^b^	8.84 ± 3.13 ^ab^	5.56 ± 1.83 ^b^
∑n-6	968.6 ± 228.3 ^b^	1086 ± 372 ^b^	2962 ± 747 ^a^	701.6 ± 202.0 ^b^	886.5 ± 198.0 ^b^	607.1 ± 336.7 ^b^
∑n-6/∑n-3	4.51 ± 0.57 ^a^	4.75 ± 1.04 ^a^	2.11 ± 1.21 ^b^	4.04 ± 0.97 ^ab^	3.83 ± 0.56 ^ab^	4.42 ± 0.95 ^a^
total	10884 ± 1123 ^b^	12131 ± 1055 ^b^	28561 ± 9741 ^a^	9141 ± 832 ^b^	8220 ± 1771 ^b^	6911 ± 1281 ^b^
AI	0.49 ± 0.11	0.52 ± 0.05	0.58 ± 0.06	0.46 ± 0.03	0.53 ± 0.21	0.41 ± 0.05
TI	0.98 ± 0.11 ^a^	0.96 ± 0.10 ^a^	0.50 ± 0.11 ^b^	1.04 ± 0.15 ^a^	1.10 ± 0.39 ^a^	0.99 ± 0.13 ^a^

Data are shown as the average ± SD, and average values sharing the same letter were not significantly different (*p* < 0.05). *n* = 4. Fatty acid is reported as the content (μg) in meat (g); t, trans; c, cis.

**Table 4:** Odor activity value (ratio) of volatilomes in meat parts from Liangshan Semi-fine Wool Sheep.

Volatilome	Threshold (μg/kg)	Neck	Chuck Roll	Thin Flank	Outside Flat	Eye of Round	Hind Shank
Alcohol							
2,3-Butanediol	95.1	—	—	—	2.60 ± 0.06 ^a^	—	0.35 ± 0.01 ^b^
1-Hexanol	5.6	17.96 ± 16.68 ^b^	17.34 ± 15.59 ^b^	69.41 ± 1.12 ^a^	9.90 ± 4.27 ^b^	31.44 ± 16.72 ^b^	26.79 ± 0.15 ^b^
1-Heptanol	5.4	—	—	5.23 ± 0.26 ^b^	3.68 ± 0.70 ^c^	—	9.23 ± 0.27 ^a^
1-Octen-3-ol	1.5	246.9 ± 245.8	214.2 ± 205.1	625.2 ± 498.4	133.8 ± 137.0	496.1 ± 603.5	57.78 ± 24.27
(*Z*)-2-Octen-1-ol	20.0	1.47 ± 0.16 ^bc^	1.28 ± 0.02 ^c^	3.39 ± 1.72 ^a^	0.67 ± 0.14 ^c^	3.05 ± 0.05 ^ab^	1.14 ± 0.03 ^c^
1-Octanol	125.8	0.16 ± 0.15	0.11 ± 0.12	0.13 ± 0.01	0.29 ± 0.01	0.42 ± 0.41	—
Aldehyde							
Hexanal	5.0	36.35 ± 38.09 ^b^	7.24 ± 3.36 ^b^	57.74 ± 34.44 ^ab^	42.34 ± 16.21 ^b^	166.5 ± 0.61 ^a^	84.93 ± 90.74 ^ab^
Heptanal	2.8	5.90 ± 0.10 ^b^	5.20 ± 0.19 b^c^	5.99 ± 1.91 ^b^	3.55 ± 0.97 ^c^	12.83 ± 0.32 ^a^	11.05 ± 0.06 ^a^
(*E*,*E*)-2,4-Nonadienal	0.1	282.8 ± 4.68 ^a^	302.2 ± 34.65 ^a^	364.1 ± 153.0 ^a^	123.5 ± 18.10 ^b^	—	385.5 ± 12.54 ^a^
Decanal	3.0	—	—	4.41 ± 1.70	—	—	4.70 ± 0.35
Nonanal	1.1	33.34 ± 41.37	12.7 ± 6.38	91.71 ± 51.17	49.03 ± 33.18	157.4 ± 125.3	77.39 ± 84.44
Ketone							
5-methyl-2-Hexanone	62.66	—	0.26 ± 0.02 ^b^	—	0.20 ± 0.02 ^c^	—	0.32 ± 0.01 ^a^

Data are shown as the average ± SD, and average values sharing the same letter were not significantly different (*p* < 0.05); —, not detected or OAV < 0.1; *n* = 4.

## Data Availability

Data will be available on request to corresponding authors.

## Supplementary Materials

The following supporting information can be downloaded at: https://www.mdpi.com/article/10.3390/vetsci12060591/s1, Table S1: Linear regression equations for fatty acids; Table S2: Volatilomes in meat parts from Liangshan Semi-fine Wool Sheep.

## Abbreviations

The following abbreviations are used in this manuscript:

LSWS	Liangshan Semi-fine Wool Sheep
PUFA	Polyunsaturated fatty acid
MUFA	Monounsaturated fatty acid
SFA	Saturated fatty acid
TPA	Texture profile analysis
GC-MS	Gas chromatography-mass spectrometry
FAME	Fatty acids methyl ester
AI	Atherogenicity index
TI	Thrombogenicity index
OAV	Odor activity value
