# Supplementary material for: Investigation of Meat Quality, Volatilome, and Fatty Acid Composition of Meat Parts from Liangshan Semi-Fine Wool Sheep
Source: Vet Sci. 2025 Jun 16;12(6):591. doi: 10.3390/vetsci12060591 (PMC12197632; doi:10.3390/vetsci12060591)
Supplement: Supplementary file 1 [file vetsci-12-00591-s001.zip › vetsci-3622452-supplementary.pdf]

Table S1. Linear regression equations for fatty acids.

Fatty acid	RT (min)	Linear equation	r	Range (μg/mL)
C6:0	5.14	y=0.0233x + 0.000214	0.998	0.02-40
C8:0	6.17	y=0.0524x + 0.00017	0.996	0.02-40
C10:0	7.41	y=0.0815x + 0.000176	0.995	0.01-20
C11:0	8.11	y=0.0839x + 0.000154	0.991	0.02-40
C12:0	8.88	y=0.0821x + 0.000393	0.993	0.01-20
C13:0	9.75	y=0.0737x + 7.5e-05	0.996	0.02-20
C14:0	10.75	y=0.0718x + 0.000586	0.991	0.02-40
C14:1T	11.4	y=0.0314x + 0.00564	0.994	0.25-20
C14:1	11.69	y=0.0302x + 0.00197	0.997	0.1-40
C15:0	11.9	y=0.0645x + 0.000212	0.996	0.02-20
C15:1T	12.6	y=0.0313x + 0.00212	0.996	0.05-20
C15:1	12.91	y=0.0289x + 0.00258	0.995	0.1-40
C16:0	13.13	y=0.0617x + 0.000192	0.992	0.03-60
C16:1T	13.84	y=0.0228x + 0.00651	0.99	0.01-20
C16:1	14.09	y=0.0222x + 0.005	0.995	0.1-40
C17:0	14.55	y=0.0534x - 8.27e-05	0.993	0.03-30
C17:1T	15.24	y=0.0228x + 0.00118	0.997	0.1-40
C17:1	15.55	y=0.0225x + 0.00316	0.994	0.02-40
C18:0	16.01	y=0.0479x + 0.000144	0.993	0.02-20
C18:1N12T	16.61	y=0.0138x + 0.00285	0.992	0.02-40
C18:1N9T	16.68	y=0.0195x + 0.00264	0.996	0.03-60
C18:1N7T	16.78	y=0.0211x + 0.00208	0.993	0.05-10
C18:1N12	16.88	y=0.0146x + 0.00222	0.995	0.1-40
C18:1N9C	16.98	y=0.0115x + 0.0026	0.992	0.02-1
C18:1N7	17.1	y=0.0221x + 0.0035	0.99	0.02-40
C18:2N6T	17.81	y=0.023x + 0.00177	0.993	0.02-20
C19:1N12T	18.18	y=0.0399x + 0.0037	0.997	0.01-5
C19:1N9T	18.3	y=0.0207x + 0.00352	0.995	0.02-40
C18:2N6	18.45	y=0.0226x + 0.0013	0.992	0.03-30
C20:0	19.2	y=0.0378x + 4.85e-05	0.992	0.02-20
C18:3N6	19.6	y=0.0179x + 0.000577	0.992	0.03-30
C20:1T	19.95	y=0.0171x + 0.00217	0.994	0.05-20
C20:1	20.26	y=0.0173x + 0.00164	0.993	0.02-20
C18:3N3	20.31	y=0.0221x + 0.00053	0.992	0.03-30
C21:0	21.08	y=0.0287x + 7.32e-05	0.991	0.02-10
C20:2	22.18	y=0.0145x + 0.00115	0.991	0.03-15
C22:0	23.25	y=0.0222x + 0.000118	0.99	0.01-10
C20:3N6	23.7	y=0.0128x + 0.000689	0.995	0.1-20
C22:1N9T	24.28	y=0.0119x + 0.00149	0.992	0.01-20
C22:1N9	24.74	y=0.0182x + 0.000603	0.992	0.1-40

C20:3N3	24.75	y=0.0154x + 0.000579	0.991	0.02-10
C20:4N6	25.03	y=0.0114x + 0.000609	0.992	0.03-15
C23:0	25.94	y=0.0186x + 0.000102	0.992	0.02-40
C22:2	27.36	y=0.0118x + 0.000701	0.994	0.1-5
C20:5N3	28.02	y=0.0173x + 0.000692	0.991	0.02-10
C24:0	28.4	y=0.0222x + 0.000247	0.992	0.01-0.5
C24:1	29.15	y=0.0216x + 0.00296	0.994	0.05-20
C22:4	29.41	y=0.0246x + 0.000765	0.994	0.02-10
C22:5N6	29.95	y=0.021x + 0.000915	0.994	0.02-5
C22:5N3	30.59	y=0.023x + 0.000986	0.995	0.02-5
C22:6N3	31.07	y=0.048x + 0.00264	0.994	0.02-40

[1] RT，Retention Time；r, correlation coefficient.

Table S2. Volatilomes in meat parts from Liangshan Semi-fine Wool Sheep.

Volatilome	Neck	Chuck Roll	Thin Flank	Outside Flat	Eye Round	Hind Shank
**Alcohols**						
(S)-6-methyl-1-octanol	3.78±0.44^{d}	2.70±0.27^{d}	13.88±6.29^{b}	7.36±0.54^{c}	20.04±1.91^{a}	—
2,3-butanediol	4.72±0.46^{c}	6.18±0.84^{c}	—	247.7±5.72^{a}	—	32.98±1.30^{b}
1-Hexanol	—	—	388.7±6.25^{a}	55.46±23.92^{c}	176.1±93.62^{b}	153.1±6.27^{bc}
1-Heptanol	—	—	28.25±1.41^{b}	19.85±3.77^{c}	—	49.83±1.44^{a}
1-Octen-3-ol	—	411.4±305.4	1210±628.2	107.3±105.02	744.2±905.3	86.67±36.41
2-Ethyl-1-hexanol	37.08±22.4^{c}	37.47±22.63^{c}	3122±292.5^{a}	862.6±466.9^{b}	478.7±151.5^{bc}	481.5±58.72^{bc}
2-Propyl-1-pentanol	26.24±1.26^{c}	25.41±2.91^{c}	103.1±2.53^{a}	96.19±7.56^{a}	—	71.35±1.39^{b}
(Z)-2-Octen-1-ol	29.49±3.16^{c}	25.51±0.46^{cd}	83.98±15.20^{a}	13.46±2.85^{d}	60.97±0.94^{b}	22.80±0.62^{cd}
1-Octanol	26.06±17.39^{ab}	5.97±4.41^{c}	15.77±0.91^{bc}	36.05±0.86^{a}	—	2.96±0.17^{c}
**Aldehydes**						
Hexanal	—	36.19±16.82^{d}	368.2±80.91^{b}	211.7±81.06^{c}	847.7±30.89^{a}	—
Heptanal	16.53±0.29^{b}	14.56±0.54^{bc}	16.77±5.35^{b}	9.93±2.72^{c}	35.92±0.90^{a}	30.47±0.94^{a}
(E,E)-2,4-Nonadienal	28.28±0.47^{b}	30.22±3.46^{b}	43.56±6.64^{a}	12.35±1.81^{c}	—	38.55±1.25^{a}
Decanal	—	—	15.37±3.38	—	—	14.11±1.06
Nonanal	14.22±9.04^{b}	13.97±7.02^{b}	124.6±37.18^{ab}	71.91±7.68^{b}	229.1±98.36^{a}	39.40±19.82^{b}
**Ketones**						
5-Methyl-2-hexanone	16.52±0.12^{b}	16.02±1.33^{b}	17.50±0.64^{b}	12.49±1.40^{c}	—	20.26±0.86^{a}
**Esters**						
Octyl formate	—	—	115.1±18.29^{a}	16.08±3.54^{c}	—	43.94±4.97^{b}
**Ethers**						
1-(pentyloxy)-Hexane	—	5.38±0.57^{b}	16.57±1.05^{a}	—	1.44±0.23^{c}	4.78±1.14^{b}
**Hydrocarbons**						

Octane	17.51±0.54^{c}	16.23±1.26^{c}	65.76±8.53^{a}	12.31±0.40^{c}	53.20±2.42^{b}	2.05±0.06^{d}
2,3,5-trimethyl-Hexane	4.26±0.11^{b}	5.35±0.09^{b}	—	—	23.42±1.27^{a}	—
2,4-dimethyl-Heptane	—	10.16±3.86^{c}	—	33.82±1.80^{b}	64.86±16.55^{a}	—
2,6-dimethyl-Nonane	25.68±18.28	17.89±2.93	—	—	—	—
2,5,6-trimethyl-Decane	—	—	12.43±7.13	11.57±1.91	—	—
4,5-dimethyl-Nonane	36.48±0.97^{b}	55.55±19.51^{b}	—	—	92.03±1.69^{a}	—
2,4,6,8-tetramethyl-1-Undecene	—	—	6.54±0.42^{a}	3.27±0.56^{b}	—	—
2,4-dimethyl-Decane	16.12±6.30^{b}	14.88±0.76^{b}	15.20±10.67^{b}	64.05±46.75^{b}	157.29±26.51^{a}	20.07±13.41^{b}
2,3,4-trimethyl-Hexane	—	—	5.33±1.39^{b}	—	29.66±1.56^{a}	—
3,4,5,6-tetramethyl-Octane	19.51±1.19^{a}	—	—	—	2.68±0.47^{b}	—
2,4,6-trimethyl-Octane	6.35±0.58^{c}	8.09±0.60^{c}	11.22±0.58^{b}	7.23±0.19^{c}	—	26.21±2.43^{a}
2,5,9-trimethyl-Decane	7.16±0.94^{a}	8.16±0.89^{a}	—	—	—	—
6-ethyl-2-methyl-Decane	10.72±0.49^{b}	12.48±1.16^{a}	—	—	—	—
3-ethyl-3-methyl-Decane	—	—	10.66±0.83^{a}	6.97±0.74^{b}	—	—
4,6-dimethyl-Dodecane	15.49±3.79	19.72±1.12	—	—	—	—
**Other**						
1-chloro-3-methyl-Butane	—	32.66±1.46^{b}	41.68±1.35^{a}	—	—	30.54±1.19^{b}
4-hydroxy-Butanoic acid	9.55±0.19^{c}	12.76±2.14^{c}	—	97.21±5.45^{b}	137.4±2.09^{a}	—
2-(1,1-dimethylethyl)-3-methyl-Oxirane	—	38.40±1.30^{d}	344.29±152.68^{bc}	156.32±45.63^{cd}	718.73±169.59^{a}	421.93±8.57^{b}
(3-methylbutyl)-Oxirane	3.77±0.28^{b}	2.54±0.35^{b}	14.24±1.46^{a}	—	—	—
(1-methylbutyl)-Oxirane	40.67±1.11^{b}	40.70±0.93^{b}	19.54±1.54^{c}	—	108.7±0.92^{a}	—
2,4-dimethyl-1-Decene	7.33±0.47^{b}	6.07±0.81^{b}	—	—	13.34±1.09^{a}	—

[1] Data were shown as average±SD and average values sharing the same letter were not significantly different ($P < 0.05$); —, not detected; n = 4.
